# The regulation of aggrecanase ADAMTS-4 expression in human Achilles tendon and tendon-derived cells

**DOI:** 10.1016/j.matbio.2008.02.002

**Published:** 2008-06

**Authors:** Anthony N. Corps, Gavin C. Jones, Rebecca L. Harrall, Valerie A. Curry, Brian L. Hazleman, Graham P. Riley

**Affiliations:** a Rheumatology Research Unit, Addenbrooke's Hospital, Cambridge, UK; b School of Biological Sciences, University of East Anglia, Norwich, UK

**Keywords:** ADAMTS, A Disintegrin And Metalloproteinase with ThromboSpondin motifs, Ct, threshold cycle, DMEM, Dulbecco's modified Eagle's medium, EGF, epidermal growth factor, FCS, fetal calf serum, FGF, fibroblast growth factor, GAPDH, glyceraldehyde 3-phosphate dehydrogenase, IL-1β, interleukin-1β, PDGF, platelet-derived growth factor, TGF-β, transforming growth factor-β, TNF, tumour necrosis factor, ADAMTS, Aggrecanase, Tendon, Transforming growth factor-β

## Abstract

Several members of the ADAMTS (A Disintegrin And Metalloproteinase with ThromboSpondin motifs) family have been identified as aggrecanases, whose substrates include versican, the principal large proteoglycan in the tendon extracellular matrix. We have characterized the expression of ADAMTS-4 in human Achilles tendon and tendon-derived cells. ADAMTS-4 mRNA levels were higher in ruptured tendon compared with normal tendon or chronic painful tendinopathy. In tissue extracts probed by Western blotting, mature ADAMTS-4 (68 kDa) was detected only in ruptured tendons, while processed ADAMTS-4 (53 kDa) was detected also in chronic painful tendinopathy and in normal tendon. In cultured Achilles tendon cells, transforming growth factor-β (TGF-β) stimulated ADAMTS-4 mRNA expression (typically 20-fold after 24 h), while interleukin-1 induced a smaller, shorter-term stimulation which synergised markedly with that induced by TGF-β. Increased levels of immunoreactive proteins consistent with mature and processed forms of ADAMTS-4 were detected in TGF-β-stimulated cells. ADAMTS-4 mRNA was expressed at higher levels by tendon cells in collagen gels than in monolayer cultures. In contrast, the expression of ADAMTS-1 and -5 mRNA was lower in collagen gels compared with monolayers, and these mRNA showed smaller or opposite responses to growth factors and cytokines compared with that of ADAMTS-4 mRNA. We conclude that both ADAMTS-4 mRNA and ADAMTS-4 protein processing may be differentially regulated in normal and damaged tendons and that both the matrix environment and growth factors such as TGF-β are potentially important factors controlling ADAMTS aggrecanase activities in tendon pathology.

## 1 Introduction

Tendons such as the Achilles are susceptible to chronic painful degenerative tendinopathy (Åström and Rausing, 1995) and ‘spontaneous’ ruptures in the absence of previous symptoms (Waterston et al., 1997). A combination of histological, biochemical and molecular biological studies have indicated that there is a normal balance between the synthesis and degradation of tendon matrix components, which is disrupted in chronic or acute tendon damage (Kannus and Jozsa, 1991; Jarvinen et al., 1997; Movin et al., 1997; Maffulli et al., 2000; Riley et al., 1994, 2002; Riley, 2004). For example, there are multiple differences in gene expression in degenerate Achilles tendons compared to normal tendons, including the expression of extracellular matrix components and the enzymes that degrade them (Ireland et al., 2001; Corps et al., 2006; Jones et al., 2006).

Subfamily M12B of the metalloproteinases (http://merops.sanger.ac.uk/) comprises the ADAM (A Disintegrin And Metalloproteinase) and ADAMTS (A Disintegrin And Metalloproteinase with ThromboSpondin motifs) enzymes. ADAMTS-1, -4 and -5 have been characterised as aggrecanases which cleave aggrecan at specific sites (Caterson et al., 2000; Kuno et al., 2000; Tang, 2001; Tortorella et al., 2001). ADAMTS-1 and -4 have also been shown to cleave versican (Sandy et al., 2001; Westling et al., 2004), which is the principal large proteoglycan expressed in mid-tendon (Robbins and Vogel, 1994; Benjamin and Ralphs, 1998; Waggett et al., 1998). In addition, ADAMTS-8, -9 and -15 have been reported to cleave aggrecan (Somerville et al., 2003; Collins-Racie et al., 2004). Cleavage products consistent with ADAMTS-specific activity are observed in cartilage and synovium explant systems and *in vivo* (Ilic et al., 1992; Caterson et al., 2000; Sandy et al., 2001; Tortorella et al., 2001; Vankemmelbeke et al., 2001; Patwari et al., 2005) and in bovine flexor tendons (Rees et al., 2000; Samiric et al., 2004), but no data have been reported for human tendons.

Using relative quantitative RT-PCR, we recently screened RNA samples from normal Achilles tendons and from individuals suffering either chronic tendon pain or spontaneous tendon rupture, for mRNA encoding all the known matrix metalloproteinases, ADAMTS and tissue inhibitors of metalloproteinases (TIMP) (Jones et al., 2006). In that screen, mRNA encoding the aggrecanases ADAMTS-1, -4, -5, -8, -9 and -15 were each detected. Of these mRNA, ADAMTS-1 mRNA was expressed at highest levels but showed little difference between normal, painful and ruptured tendons, while ADAMTS-4 mRNA showed the largest change, increasing 8-fold in ruptured tendons (Jones et al., 2006). Previous studies have highlighted the potential role of growth factors such as platelet-derived growth factor (PDGF) and transforming growth factor-β (TGF-β) in tendon pathology (Fenwick et al., 2001; Rolf et al., 2001), but little is known about their effect on aggrecanases. In addition, the mechanical environment acting on tenocytes cultured in a three-dimensional matrix has been shown to regulate some metalloproteinase activities (Lavagnino and Arnoczky, 2005). We have now tested the effects of growth factors on the regulation of ADAMTS-4 mRNA expression in Achilles tendon-derived cells in both monolayer and three-dimensional collagen gel cultures, and examined the expression of ADAMTS-4 protein in tendon tissues and cells.

## 2 Results

### 2.1 Expression of ADAMTS-4 mRNA and protein in tendon tissue samples

**Fig. 1:**
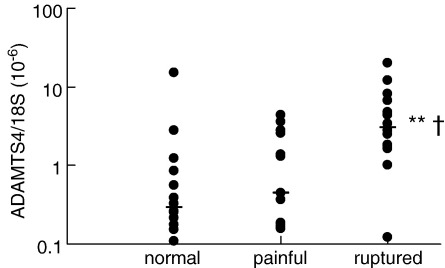
ADAMTS-4 mRNA expression in normal, painful and ruptured tendon. Tendon RNA samples were analysed for ADAMTS-4 mRNA, expressed relative to 18S rRNA as outlined in the text. Each point represents an individual sample. The median values are shown (horizontal bar). ** *P* < 0.01, † *P* < 0.05, for differences between ruptured tendon and the normal and painful tendon groups respectively, by Wilcoxon rank sum test.

We first confirmed the results of our previous screen of tendon tissue RNA (Jones et al., 2006), using an extended sample set (*n* = 14 per group) and a second, independently-derived, relative quantitative real-time RT-PCR primer-probe set, which was also used to determine ADAMTS-4 mRNA expression in the cell studies described below. ADAMTS-4 mRNA expression was detected in all tendon tissue samples. Normal, painful and ruptured tendon samples showed median values of ADAMTS-4 mRNA expression, relative to 18S rRNA, of 0.29 × 10^− 6^, 0.45 × 10^− 6^ and 3.07 × 10^− 6^ respectively (Fig. 1). The ruptured group differed significantly from the normal and painful groups (*P* < 0.01 and *P* < 0.05, respectively, by Wilcoxon rank sum test).

**Fig. 2:**
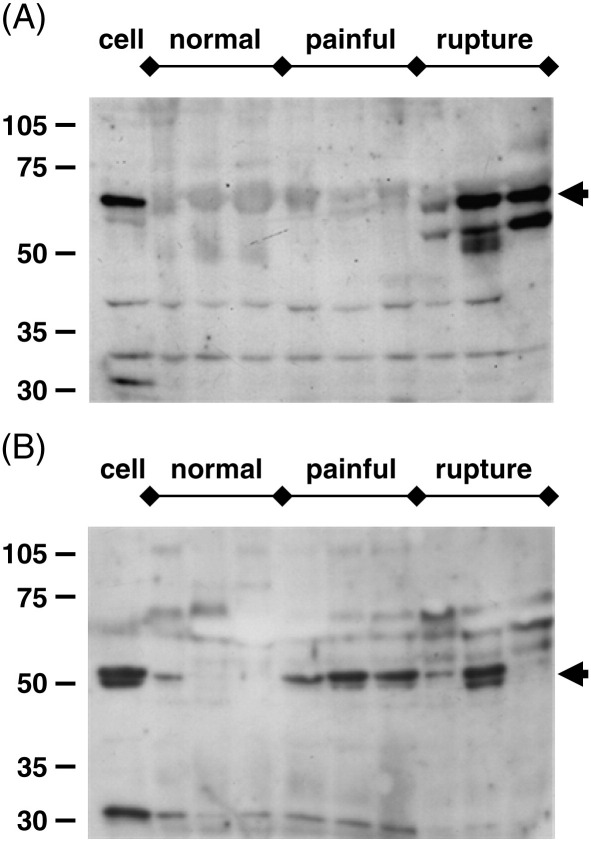
ADAMTS-4 immunoreactivity in tendon tissue. Western blots of protein extracts (10 μg/lane) from 3 samples each of normal, painful and ruptured tendon tissue were probed using the ADAMTS-4-specific antibodies (A) AHP821GA and (B) PA1-1749. The lane marked *cell* contained extract from a TGF-β-treated tendon cell culture. The *arrows* indicate the mature and processed forms of the ADAMTS proteins discussed in the text. Similar results were obtained using 3 further samples per tissue group, run on a separate pair of blots.

To assess the levels and processing of ADAMTS-4 protein in tendon tissue, we extracted samples using a neutral extraction buffer containing NP-40 (Gao et al., 2002). The major band detected by antibody AHP821GA (raised against a C-terminal domain peptide) was consistent with the mature 68 kDa form of ADAMTS-4 (Fig. 2A), while the major band detected by antibody PA1-1749 (raised against a catalytic domain peptide) was consistent with the processed 53 kDa form of ADAMTS-4 (Fig. 2B). The 68 kDa form of ADAMTS-4 was detected readily in ruptured tendon tissue, but little or none was detected in normal or painful tendon samples (Fig. 2A and data not shown). The 53 kDa form was detected in only three of the six samples of normal tendon tested, and to a variable extent in ruptured tendon samples, but was readily detected in all samples of painful tendon (Fig. 2B and data not shown). The detection of the two principal forms of ADAMTS-4 by these two antibodies was also observed in samples of cultured cells extracted using the NP-40-containing buffer (Fig. 2, lane marked cell).

The preference of the PA1-1749 antibody for the 53 kDa form of ADAMTS-4 (Fig. 2B) is in agreement with studies using cartilage extracts (Gao et al., 2002; Patwari et al., 2005). Several additional faint bands were present across all lanes and may represent non-specific cross-reactivity (Fig. 2B); of these, a band of about 70 kDa prevented the unequivocal detection of the 68 kDa form of ADAMTS-4 by this antibody. The preference of the C-terminal domain antibody AHP821GA for the 68 kDa form of ADAMTS-4 (Fig. 2A) is similar to that of an antibody raised against the cysteine-rich region of ADAMTS-4 used by Patwari et al. (2005), although the precise epitope of AHP821GA within the cysteine-rich and spacer regions was not defined by the supplier. An additional band of between 55 and 60 kDa was detected by the antibody AHP821GA, principally in those samples with high expression of the 68 kDa form (Fig. 2A); this may represent an additional or alternative cleavage of the 68 kDa form in the spacer domain.

### 2.2 Regulation of ADAMTS-4 mRNA expression in cultured tendon cells

**Fig. 3:**
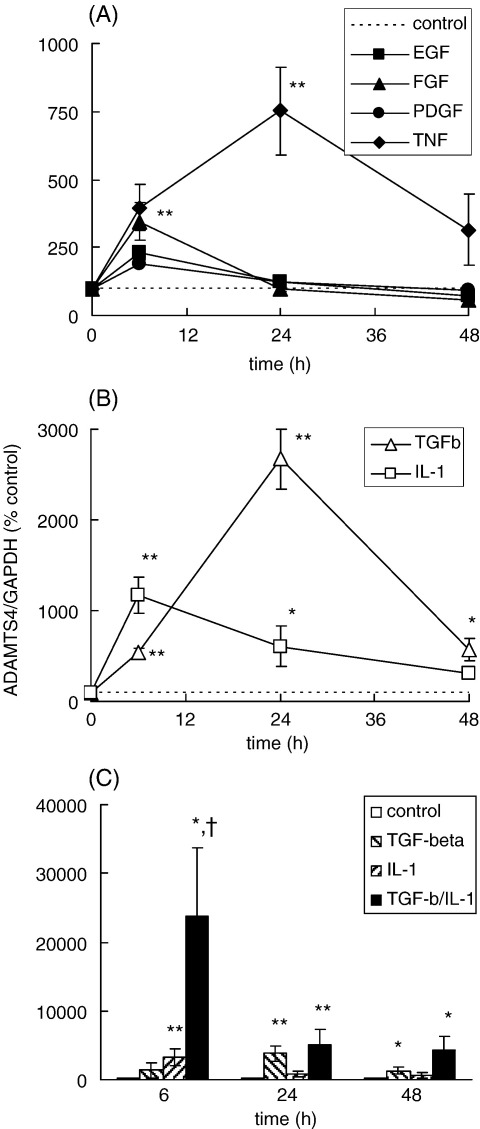
Regulation of ADAMTS-4 mRNA expression in cultured tendon cells by growth factors and cytokines. Tendon cells were incubated for 6, 24 or 48 h with EGF (10 ng/ml, ■), FGF (1 ng/ml, ▲), PDGF (10 ng/ml, ●), TNF (10 ng/ml, ♦), IL-1β (1 ng/ml, □), TGF-β (1 ng/ml, Δ) or TGF-β plus IL-1β (panel C, filled column). Total cell RNA was extracted and analysed for ADAMTS-4 mRNA, expressed relative to GAPDH mRNA and normalised to the value obtained for control cells at the same time-point (defined as 100% and shown as a broken line). Results are mean ± s.e.m. from at least 4 experiments with each agonist (panels A,B) or 3 experiments (panel C); * *P* < 0.05, ** *P* < 0.01 compared with the control at the same time point, determined from 95% or 99% confidence intervals of the treated samples; † *P* < 0.05 for TGF-β plus IL-1β compared with either cytokine alone.

**Fig. 4:**
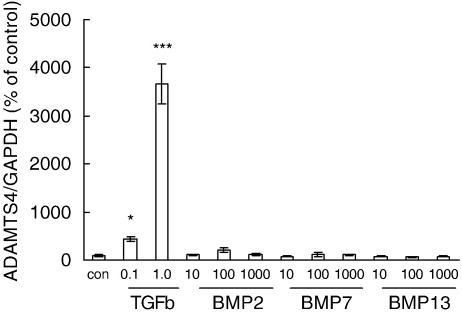
Regulation of ADAMTS-4 mRNA expression in cultured tendon cells by TGF-β and BMP. Tendon cells were incubated for 24 h with TGF-β or BMP-2, -7 or -13 at the concentrations shown (ng/ml). Total cell RNA was extracted and analysed for ADAMTS-4 mRNA, expressed relative to GAPDH mRNA and normalised to the mean value obtained for control cells (defined as 100%). Results shown are mean ± s.e.m. of three wells per treatment in a single experiment; * *P* < 0.05, *** *P* < 0.001 by t-test. Similar results were obtained in a separate experiment with cells derived from a second donor.

Tendon cells in culture expressed each of ADAMTS-4, ADAMTS-1 and ADAMTS-5 mRNA. When the cells were rinsed into fresh serum-free medium, ADAMTS-4 mRNA levels in control cells showed a decrease during the first 48 h incubation, but subsequently showed only small (less than 2-fold) variations that were not consistent between experiments (data not shown). Growth factors and cytokines, added to the cells after 48 h adaptation to serum-free medium, stimulated the expression of ADAMTS-4 mRNA, with different characteristic time-course and magnitude (Figs. 3 and 4). Epidermal growth factor (EGF), fibroblast growth factor (FGF) or PDGF each induced small transient stimulations of ADAMTS-4 mRNA levels (about 3-fold at 6 h; Fig. 3A). Stimulation by interleukin-1β (IL-1β) followed a similar time-course but was greater in magnitude, reaching more than 10-fold (Fig. 3B), while stimulation by tumour necrosis factor (TNF) showed a significantly longer time-course, increasing between 6 and 24 h to 8-fold (Fig. 3A).

The greatest stimulation of ADAMTS-4 mRNA expression by an individual growth factor or cytokine was induced by TGF-β (Figs. 3 and 4). Detectable stimulation was obtained using 0.1 ng/ml TGF-β and maximum stimulation (typically more than 20-fold) occurred after 24 h with 1–10 ng/ml TGF-β. The stimulation of ADAMTS-4 mRNA by TGF-β showed a marked synergy with that induced by IL-1β (Fig. 3C); the combination of TGF-β with IL-1β induced substantially higher levels of ADAMTS-4 mRNA over the early period when IL-1β was active individually (6–12 h), but by 24 h the combined stimulation was not reproducibly different from that achieved using TGF-β alone (Fig. 3C). Other members of the TGF-β superfamily, the bone morphogenetic proteins (BMP), have been implicated in tendon damage and repair (Aspenberg and Forslund, 2000; Neuwirth et al., 2006). We therefore tested BMP-2, BMP-7 and BMP-13 for effects on tendon cell mRNA expression. None of these BMP stimulated ADAMTS-4 mRNA expression, compared with 10- to 30-fold stimulation by TGF-β (Fig. 4). However, both BMP-2 and BMP-13 stimulated the expression of aggrecan mRNA between 4- and 14-fold in the same cell samples (data not shown), indicating that active receptor signalling occurred over the dose range used.

### 2.3 Expression of ADAMTS-4 protein in tendon cells

**Fig. 5:**
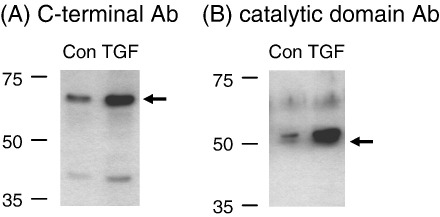
ADAMTS-4 protein expression in cultured tendon cells. Western blots of protein extracts from tendon cell cultures incubated for 24 h with vehicle (Con) or TGF-β (1 ng/ml) were probed using the ADAMTS-4-specific antibodies (A) AHP821GA and (B) PA1-1749. The *arrows* indicate the mature (68 kDa) and processed (53 kDa) forms of ADAMTS-4 discussed in the text. Similar results were obtained in two additional experiments.

Tendon cell protein extracts, prepared by lysis in the same NP-40-containing extraction buffer used for tendon tissue samples, contained ADAMTS-4-immunoreactive bands consistent with the mature (68 kDa) and processed (53 kDa) forms of the enzyme (Figs. 2 and 5). Both of these major forms showed an increase in intensity in cells treated for 24 h with TGF-β (Fig. 5). In some experiments, the increase could be detected at 6 or 12 h, but by 48 h there was no reproducible difference in the intensity with/without TGF-β (data not shown). In contrast to TGF-β, IL-1β did not reproducibly increase the levels of ADAMTS-4 proteins in cell extracts at 6–12 h (data not shown), i.e. at the time when IL-1β stimulated ADAMTS-4 mRNA (Fig. 3B). This may be related to the lower level of mRNA stimulated by IL-1β than by TGF-β (Fig. 3B), but it is also possible that IL-1β may also stimulate ADAMTS-4 protein turnover; it has been reported that IL-1 can alter ADAMTS-4 processing in cartilage explants (Patwari et al., 2005; see also Discussion).

### 2.4 Comparison of cellular ADAMTS-1 and ADAMTS-5 mRNA expression with that of ADAMTS-4 mRNA

**Fig. 6:**
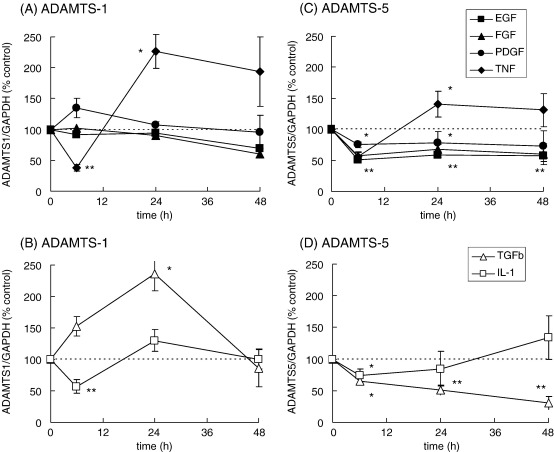
Regulation of ADAMTS-1 and ADAMTS-5 mRNA expression in cultured tendon cells. Tendon cells were incubated with growth factors and cytokines, and total cell RNA was analysed for ADAMTS-1 or ADAMTS-5 mRNA, as described in the legend to Fig. 3. Results are mean ± s.e.m. from at least 4 experiments with each agonist; * *P* < 0.05, ** *P* < 0.01 compared with the control at the same time point, determined from the 95% or 99% confidence intervals of the treated samples.

We noted previously (Jones et al., 2006) that ADAMTS-1 mRNA expression showed little variation between tendon tissue sample groups while ADAMTS-5 mRNA decreased in painful tendons, thus contrasting with the pattern of ADAMTS-4 mRNA expression. In the present study, growth factors and cytokines had differential effects on ADAMTS-1 or ADAMTS-5 mRNA expression (Fig. 6) compared with that of ADAMTS-4 (Fig. 3). None of EGF, FGF and PDGF had a significant effect on ADAMTS-1 mRNA expression (Fig. 6A), but each induced significant decreases in ADAMTS-5 mRNA levels (*P* < 0.01, except for PDGF *P* < 0.05), which were maintained over 48 h (Fig. 6C). The addition of TNF or IL-1β induced a transient decrease of ADAMTS-1 mRNA expression: a significant decrease at 6 h (64 ± 4% by TNF, 55 ± 5% by IL-1β), was followed by a return to control levels or higher at 24 h (Fig. 6A,B). Similarly, TNF and IL-1β induced a transient decrease of ADAMTS-1 mRNA expression (Fig. 6C,D). Finally, TGF-β had contrasting effects on these two mRNA, inducing a small (2- to 3-fold) transient stimulation of ADAMTS-1 mRNA expression at 24 h (Fig. 6B) and a persistent decrease of ADAMTS-5 mRNA (Fig. 6D).

### 2.5 Cellular ADAMTS mRNA expression in three-dimensional collagen gel cultures

**Fig. 7:**
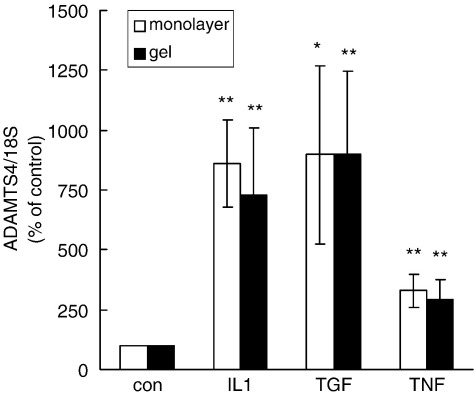
Regulation of ADAMTS-4 mRNA expression in tendon cells cultured in collagen gels. Tendon cells in monolayer cultures (open columns) or collagen gels (filled columns) were incubated for 24 h with vehicle (Con), IL-1β (1 ng/ml), TGF-β (1 ng/ml) or TNF (10 ng/ml). Total cell RNA was extracted and analysed for ADAMTS-4 mRNA, expressed relative to 18S rRNA and normalised to the value obtained for control cells in monolayer culture or gel respectively (defined as 100%): note that the mean control level in collagen gels was 2.6-fold higher than that in monolayer cultures. Results shown are mean ± s.e.m. from 4 experiments; * *P* < 0.05, ** *P* < 0.01 compared with the respective control values, determined from the 95% or 99% confidence intervals of the treated samples.

When cells were placed in three-dimensional collagen gel cultures, ADAMTS-4 mRNA levels increased compared with those in parallel monolayer cultures (Table 1). In further contrast with ADAMTS-4, levels of both ADAMTS-1 and ADAMTS-5 mRNA were lower in cells in collagen gel cultures compared with monolayers (Table 1). These differences were established over the first 48 h of serum-free culture in collagen gel or monolayer (Table 1) and then persisted for up to 7 days without significant change (data not shown). Each of IL-1β, TGF-β and TNF were able to stimulate ADAMTS-4 mRNA expression above the raised basal level in collagen gels (Fig. 7), indicating that there was no general mechanistic interference between the cytokine-induced and gel-induced stimuli.

## 3 Discussion

Various studies have indicated that there is a balance between the synthesis and breakdown of matrix components in normal tendons, and that this balance is disrupted in tendinopathies (see Introduction). In this paper we have shown that mRNA encoding the aggrecanase ADAMTS-4 is regulated independently from ADAMTS-1 or ADAMTS-5 mRNA in both human Achilles tendon tissue samples and cultured tendon cells, and that mature and processed forms of ADAMTS-4 protein may be detected in tendon tissue and cellular extracts.

Independent regulation of the ADAMTS aggrecanase genes has been noted in several tissues (Flannery et al., 2000; Bau et al., 2002; Pratta et al., 2003; Kevorkian et al., 2004; Moulharat et al., 2004; Wachsmuth et al., 2004; Cross et al., 2005). Our studies indicate that both the biomechanical environment and various cytokines and growth factors can contribute differential regulatory effects to ADAMTS gene expression in tendon cells. Each of EGF, FGF and PDGF increased ADAMTS-4 and decreased ADAMTS-5 mRNA expression respectively, which is the first demonstration of such effects of these growth factors. Several previous studies have described effects of IL-1β on ADAMTS expression in cells or explants from different tissues, but with considerable variation in the degree of stimulation observed (Caterson et al., 2000; Tortorella et al., 2001; Vankemmelbeke et al., 2001; Koshy et al., 2002; Yaminishi et al., 2002; Tzusaki et al., 2003; Wachsmuth et al., 2004; Patwari et al., 2005). The only previous study using human tendon cells reported small, variable, effects of IL-1β on ADAMTS-4 mRNA expression, measured after 16 h (Tzusaki et al., 2003): our work indicates that the optimum stimulation by IL-1β occurs at an earlier time (6 h—Fig. 3). In contrast, there was a marked transient decrease in ADAMTS-1 and ADAMTS-5 mRNA expression after this time of treatment with either IL-1β or TNF (Fig. 6).

TGF-β induced the largest stimulation of ADAMTS-4 mRNA expression by any individual growth factor, and co-addition of TGF-β with IL-1β gave a marked synergy (Fig. 3), which has not been described previously. TGF-β also induced a small stimulation of ADAMTS-1 mRNA expression but decreased that of ADAMTS-5 (Fig. 6). Contrasting effects of TGF-β on different ADAMTS mRNA have been reported previously in synoviocytes (Yaminishi et al., 2002), chondrocytes (Moulharat et al., 2004) and prostate stromal cells (Cross et al., 2005). We also tested several BMP, members of the TGF-β superfamily which have been implicated in tendon damage and repair (Aspenberg and Forslund, 2000; Neuwirth et al., 2006) and which act through related receptors to activate different SMAD transcription factors from TGF-β (Shi and Massague, 2003). However, none of these BMP had effects similar to those of TGF-β on ADAMTS-4 mRNA (Fig. 4). The activation of ADAMTS-4 expression thus shows a degree of specificity within the TGF-β superfamily-SMAD signalling network.

In fetal bovine tendon, TGF-β has been implicated in mediating the effects of compressive loading on matrix gene expression (Robbins et al., 1997). Indeed, mechanical loading of tendon is a potential regulatory mechanism for metalloproteinase gene expression (Banes et al., 1999; Lavagnino et al., 2003; Lee et al., 2005) that might complement the regulation of ADAMTS expression by growth factors and cytokines. Mechanotransduction signals are mediated, at least in part, by interactions of cells with the three-dimensional environment of the extracellular matrix (Garvin et al., 2003; Lavagnino and Arnoczky, 2005). We found that placing tendon cells in a three-dimensional collagen gel (a version of the fibroblast-populated collagen lattice: Ehrlich and Rittenberg, 2000; Grinnell, 2003) increased the expression of ADAMTS-4 mRNA relative to the monolayer and decreased that of ADAMTS-1 and ADAMTS-5 (Table 1). At least three factors may contribute to these effects (i) a specific interaction of the cells with collagen, (ii) a response to being in three dimensions rather than two, and (iii) a response to tension within the matrix. Although further work will be required to distinguish between these and other explanations, in initial experiments we have found no reproducible difference in ADAMTS expression between cells in anchored collagen gels (as in Table 1) and parallel gels that were free-floating, having been released from the culture dish (data not shown).

Studies using explant and cell cultures from other tissues have shown increased aggrecanase activity under conditions where ADAMTS mRNA and/or protein levels are not altered (Caterson et al., 2000; Flannery et al., 2000; Yaminishi et al., 2002; Pratta et al., 2003; Patwari et al., 2005), indicating that both the translation of the mRNA and the processing of the ADAMTS proenzymes are subject to regulation. In tendon tissue extracts, we found that mature 68 kDa ADAMTS-4 immunoreactivity was principally detected in ruptured tendons, consistent with the elevation of mRNA in these samples (Figs. 1 and 2). However, in each clinical group, notably in painful tendinopathy, there were significant levels of the 53 kDa form detected preferentially by the PA1-1749 antibody (Fig. 2B). These results suggest that altered processing of ADAMTS-4 protein may occur between the different clinical groups. The principal change observed in the cellular expression of ADAMTS-4 protein *in vitro* was an increased level (of both main ADAMTS-4 forms) in cells stimulated by TGF-β (Fig. 5). In some experiments, we observed a band of 40–45 kDa (reactive with the PA1-1749 antibody) in TCA-precipitated supernatant medium from 48-h -incubated cells (data not shown). This band was significantly smaller than the 53 kDa band present in cell extracts, and was elevated in cultures treated with either TGF-β or IL-1β. It may thus represent further processing and release of ADAMTS-4 from the cells in response to these cytokines, analogous to some of the effects reported in other model systems (Patwari et al., 2005).

In conclusion, we have shown that ADAMTS-4 mRNA is regulated differentially from ADAMTS-1 and ADAMTS-5 mRNA in Achilles tendon and tendon-derived cells *in vitro*, and that active forms of ADAMTS-4 are present in the tendon tissues. The apparent functional redundancy of the ADAMTS aggrecanases is complex, as emphasised in studies of gene knockouts in mice, which indicate a major role for ADAMTS-5 aggrecanase in mouse cartilage (Stanton et al., 2005), but not ADAMTS-1 or ADAMTS-4, despite the fact that these are expressed in this tissue (Glasson et al., 2004; Little et al., 2005). However, none of these gene knockout studies addressed the question of ADAMTS aggrecanase function in tendon. Further studies will be required to determine the contribution of ADAMTS-4 to the observed changes in proteoglycans in tendinopathy.

## 4 Experimental procedures

### 4.1 Materials

Dulbecco's modified Eagle's medium (DMEM), fetal calf serum (FCS), antibiotics and oligonucleotide primers were obtained from Invitrogen (Paisley, UK). One-Step RT-PCR reagents and FAM-labelled oligonucleotide probes were obtained from Applied Biosystems (Warrington, UK) and Sigma-Genosys (Haverhill, UK). IL-1β was a gift from Glaxo Wellcome (Stevenage, UK) and aliquots (1 μg/ml) were stored at − 70 °C. Other growth factors and cytokines were obtained from R&D Systems (Abingdon, Oxford, UK) or Insight Biotechnology (Wembley, UK) and were solubilised and stored according to the suppliers' instructions. Primary anti-ADAMTS-4 antibodies were as follows: antibody AHP821GA, raised against a C-terminal domain peptide, was from Serotec (Oxford, UK); antibody PA1-1749, raised against a catalytic domain peptide (also referred to as JSCVMA: Patwari et al., 2005) was from Affinity Bioreagents (Golden, CO, USA), obtained through Cambridge Bioscience (Cambridge, UK). Horseradish peroxidase-coupled secondary antibodies were from Dako (Ely, UK).

### 4.2 Tendon specimens

All procedures had appropriate local ethical committee approval, and consent was obtained from informed patients. A detailed characterisation of the sample groups has been published (Jones et al., 2006). Normal Achilles tendon specimens were obtained from cadaver material, taken within 48 h of death, or from patients undergoing amputation of the leg for the removal of bone tumours, and showed normal histological appearance. Tissue from individuals suffering painful tendinopathy for more than 6 months was taken from the site of the lesion during surgery and showed abnormal histological appearance. Tissue from individuals undergoing repair of ruptured tendon, mostly within 48 h of the rupture occurring, was trimmed from the site of the rupture. Specimens were transported to the laboratory in ice-cold balanced salts solution, and were stored frozen at − 70 °C. The age of the individuals (14 per group) from which tissue was used for RNA analysis was as follows: normal tendon, range 20–97 years, median 55; painful tendinopathy, range 32–59 years, median 43; ruptured tendon, range 25–69 years, median 45. None of the ADAMTS mRNA expression showed a significant variation with age in this study (data not shown).

### 4.3 RNA isolation from tendon tissue samples

Total RNA was isolated from frozen tissue samples by a modified Tri-Spin protocol as described previously (Ireland et al., 2001) and was finally resuspended in 100 μl water. The concentration of RNA was estimated using a NanoDrop spectrophotometer (NanoDrop Technologies, Delaware, USA; courtesy of Prof. D.E. Neal's group at the Hutchison/MRC Research Centre, Cambridge). The majority of samples yielded between 20 and 70 ng RNA/mg wet weight, consistent with our previous experience of human tendon samples, most of which have low cellularity (Ireland et al., 2001; Jones et al., 2006). Five of the painful tendinopathy samples and three of the ruptured tendon samples gave RNA yields between 85 and 330 ng/mg, significantly above the normal range, consistent with an increase in cellularity observed in such samples (Åström and Rausing 1995; Jones et al., 2006). The RNA was diluted to 1 ng/μl, and stored at − 70 °C as aliquots which were thawed once only. Samples from each group gave well-defined bands of 28S and 18S rRNA on 1.2% (w/v) agarose gels, with no evidence of significant degradation. ADAMTS-4 mRNA was assayed by One-Step relative quantitative real-time RT-PCR, normalised to 18S rRNA, as described below.

### 4.4 Protein extracts and Western blotting

Protein was extracted from tendon tissue samples by homogenisation in 3 volumes of extraction buffer (50 mM Tris pH 7.0, 150 mM NaCl, 5 mM EDTA, 0.5% NP-40 (Gao et al., 2002) with added protease inhibitors: 10 μM E64, 1.5 μM pepstatin A and 1 mM PMSF) followed by incubation overnight at 4 °C and centrifugation (14,000 g, 4 °C, 15 min). The protein content of the clarified extracts was determined, and 50 μg of each extract was precipitated using trichloroacetic acid (3.3% w/v), washed with acetone, dried and redissolved in 100 μl of protein gel sample buffer. Ten μg (20 μl) of extract was boiled with gel sample buffer for 3 min and subjected to SDS-PAGE under reducing conditions, using a 4% (w/v) polyacrylamide stacking gel with a 10% (w/v) polyacrylamide gel. The proteins were electroblotted onto PVDF membranes, which were then blocked, incubated with primary antibodies, washed, incubated with secondary antibodies, washed again, incubated with CDP-Star (Roche, Lewes, UK) and exposed to film, all using standard methods. An aliquot of an extract from TGF-β-treated tendon cells was included on each gel (see Fig. 2, lanes marked cell) to act as a standard for comparison of the autoradiographs.

### 4.5 Culture of tendon cells

Tendon cells were isolated by outgrowth from explant cultures established from mid-tendon samples taken during surgery for chronic tendinopathy. Cells were maintained and passaged in DMEM containing 10% (v/v) FCS, penicillin, streptomycin and 25 mM HEPES, and were used between passages 4 and 10. Each experiment was performed separately using cells derived from at least three separate patients with similar results. Cells were seeded at 10^5^/well in 6-well plates and were incubated for 3 days before the experiment. They were rinsed with serum-free DMEM containing insulin, transferrin and selenium and were incubated in the same medium for 48 h. Cytokines and growth factors were then added to the medium and equivalent additions of vehicle were made to control wells. After a further incubation (6–48 h), the cells were rinsed with balanced salts solution and solubilised in TRI-Reagent (Sigma; 1 ml/well). RNA was isolated from the TRI-Reagent extracts by phenol-chloroform phase separation followed by precipitation with isopropanol and ethanol. Proteins were extracted from parallel wells using the NP-40-containing buffer described above, and the extracts were clarified by centrifugation (14,000 g, 4 °C, 15 min) and prepared for Western blotting as described above.

Cells for collagen gel cultures were centrifuged (5 min at 300 g), resuspended at 2 × 10^6^/ml in serum-free DMEM, and placed on ice. Rat-tail Type I collagen solution (in 0.6% acetic acid; First-Link, Birmingham, UK) was mixed on ice with 10 × DMEM, neutralised using NaOH, and mixed thoroughly with the cell suspension. Aliquots of the mixture, containing 1 × 10^5^ cells in 0.5 ml of collagen (1.6 mg/ml) in DMEM, were placed in the wells of a 24-well plate. The collagen was allowed to set at room temperature (about 20 min) before being overlaid with 1 ml of DMEM. Cells for parallel monolayer cultures were centrifuged, resuspended and plated in neighbouring wells at 2 × 10^4^ cells/well in DMEM containing 10% FCS to promote attachment. After attachment, the cells were rinsed and incubated in serum-free DMEM, and monolayer and gel cultures were incubated for a further 48 h before harvest in TRI-Reagent (1 ml/well for monolayers and 2 ml/well for collagen gels). RNA was isolated from the TRI-Reagent extracts as described above.

### 4.6 Relative quantitative real-time RT-PCR

One-Step real-time RT-PCR reactions were performed in a GeneAmp 5700 (Applied Biosystems). The primers and probe for glyceraldehyde 3-phosphate dehydrogenase (GAPDH) and 18S rRNA have been described previously (Ireland et al., 2001; Corps et al., 2006). Primers and probes for ADAMTS mRNA were designed using Primer Express (Applied Biosystems). Each ADAMTS amplicon was chosen to include exon–exon boundaries to prevent amplification of genomic DNA, and no signal was produced if either the RNA or the reverse transcriptase was omitted. BLASTn searches (www.ncbi.nlm.nih.gov/BLAST) revealed no significant similarity to other sequences. Each primer pair generated a single product of the appropriate size, and no signal was produced when the wrong probe was included with each primer pair, indicating an absence of cross-reactivity. Accession numbers, amplicons, forward primer (F), reverse primer (R) and probe (P) sequences were as follows:

ADAMTS-1 (NM_006988; 77 bp):
  F = CCAGTGACCGGGATGCA
  R = ACATGTCTGGGACCCACACA
  P = TATGACACAGCAATTCTTTTCACCAGACAGGAC
ADAMTS-4 (NM_005099; 83 bp):
  F = GCAACGTCAAGGCTCCTCTT
  R = CTCCACAAATCTACTCAGTGAAGCA
  P = AGACCCCGAAGAGCCAAGCGCT
ADAMTS-5 (XM_047802; 73 bp):
  F = AGGAGCACTACGATGCAGCTATC
  R = CCCAGGGTGTCACATGAATG
  P = TGCCCACATAAATCCTCCCGAGTAAACA

Standard curves were run in each assay, using freshly-diluted aliquots of pooled tendon cell or tissue RNA. For each target, this produced a linear plot of threshold cycle (Ct) against log(dilution), whose slope was within 10% of the expected value, indicating a similar, near-maximum efficiency for each target. This was confirmed by the demonstration of constant Ct differences with dilution in pairwise comparisons of each target mRNA. For each experiment, all samples of RNA were assayed in duplicate or triplicate wells on the same plate (typically 2 ng/well of total RNA for mRNA targets, and 20 pg/well of total RNA for the more abundant 18S rRNA). The values obtained for ADAMTS mRNA expression were normalised for GAPDH mRNA or 18S rRNA expression in the same sample, using the formula

$$ADAMTS/\left(normalising\;RNA\right)=2^{\left[Ct\left(normalising\;RNA\right)-Ct\left(ADAMTS\right)\right]}$$

and correcting for the different input RNA into the 18S rRNA assay. Values were compared against those for control cultures in the same experiment (i.e. cultures receiving the same changes of medium but incubated with vehicle rather than growth factors or cytokines) as 100%.

## Figures and Tables

**Table 1:** Expression of ADAMTS mRNA in tendon cells incubated for 48 h in collagen gels relative to parallel monolayer cultures

mRNA	(Gel/monolayer × 100%)	
ADAMTS-4	494 ± 144	(*P* < 0.05)
ADAMTS-1	27 ± 13	(*P* < 0.01)
ADAMTS-5	34 ± 14	(*P* < 0.01)
GAPDH	94 ± 28	(ns)

ADAMTS or GAPDH mRNA was normalised to 18S rRNA, and the value for collagen cultures was expressed relative to that in parallel monolayer cultures of the same cells (defined as 100%). Mean ± s.e.m. from experiments using cells from 5 separate patients. *P* values were determined from a one-sample *t*-test against a theoretical mean of 100%. ns = not significant.
